# Management of an affiliated physics residency program using a commercial software tool

**DOI:** 10.1120/jacmp.v11i3.2996

**Published:** 2010-06-01

**Authors:** Albert S. Zacarias, Michael D. Mills

**Affiliations:** ^1^ Department of Radiation Oncology University of Louisville School of Medicine Louisville KY 40202 USA

**Keywords:** residency program, CAMPEP, clinical training program, competency, medical physics training

## Abstract

A review of commercially available allied health educational management software tools was performed to evaluate their capacity to manage program data associated with a CAMPEP‐accredited Therapy Physics Residency Program. Features of these software tools include: a) didactic course reporting and organization, b) competency reporting by topic, category and didactic course, c) student time management and accounting, and d) student patient case reporting by topic, category and course. The software package includes features for recording school administrative information; setting up lists of courses, faculty, clinical sites, categories, competencies, and time logs; and the inclusion of standardized external documents. There are provisions for developing evaluation and survey instruments. The mentors and program may be evaluated by residents, and residents may be evaluated by faculty members using this feature. Competency documentation includes the time spent on the problem or with the patient, time spent with the mentor, date of the competency, and approval by the mentor and program director. Course documentation includes course and lecture title, lecturer, topic information, date of lecture and approval by the Program Director. These software tools have the facility to include multiple clinical sites, with local subadministrators having the ability to approve competencies and attendance at clinical conferences. In total, these software tools have the capability of managing all components of a CAMPEP‐accredited residency program. The application database lends the software to the support of multiple affiliated clinical sites within a single residency program. Such tools are a critical and necessary component if the medical physics profession is to meet the projected needs for qualified medical physicists in future years.

PACS numbers: 87.90.+y, 87.53.‐j

## I. INTRODUCTION

There are new requirements for eligibility to take the American Board of Radiology (ABR) initial certification examination in radiological physics. Beginning in 2014, in order to take the ABR Part 1 examination in Radiologic Physics, candidates must be enrolled in or have completed a CAMPEP‐accredited residency program. As of 2014, academic programs without an associated link to an accredited residency program will no longer be able to guarantee entry into the medical physics profession.^(^ ^1^ ^)^

The Commission on Accreditation of Medical Physics Education Programs (CAMPEP) was formed primarily to accredit medical physics graduate education programs and residency education programs. Presently, there are 21 CAMPEP‐accredited therapy programs in the United States, and seven in Canada. The United States programs graduate approximately 25 residents per year. The projected need for radiation therapy physicists between 2015 and 2020 is currently being researched by the Medical Physics Workforce Subcommittee of the American Association of Physicists in Medicine (AAPM) Professional Services Committee. Preliminary estimates are that between 125 and 200 therapy medical physicists will be needed per year after 2015.^(^ ^2^ ^)^ Clearly, there will be a large gap between the current number of accredited residency program graduates and the number of medical physicists required unless there are dramatic changes in the way medical physicists are educated and trained within accredited residency programs. If the profession is unable to train enough therapy medical physicists, it is likely the work week for practicing physicists will increase significantly. This may encourage more medical physicists to retire or leave the profession, having a negative impact on the quality of patient care and exacerbating the shortage of medical physicists.

In August 2008, the AAPM published the Task Group 133 Report: *Alternative Clinical Training Pathways for Medical Physicists*.^(^ ^3^ ^)^ Key options available for clinical residency training and recommendations from this report include:

- A formal two‐year residency program at an academic center offering a complete range of treatment techniques and with many, often specialized, qualified medical physicists (QMPs). Such a program, if CAMPEP‐accredited, may serve as a primary site.
- A formal two‐year residency offered at a center with more limited resources but affiliated with a CAMPEP‐accredited center.
- Incorporation of a residency program in a professional degree such as Doctor of Medical Physics where it may replace the research/project component of the more conventional Master's and Doctoral degrees. (Note: the purpose of this article is to evaluate software tools for the management of a physics clinical residency program; no evaluation was conducted to determine the suitability of these tools for a physics clinical academic program or for the academic portion of a physics professional doctorate program).

To provide the level of scheduling, tracking, evaluation and documentation expected of Graduate Medical Education (GME) clinical residency programs is a difficult task without automated and customized management tools. The complexity of management increases if residents spend the majority of their time at remote affiliated centers. How can the CAMPEP‐accredited residency program manage program data for the training at an affiliated center including tracking time in conferences, completion of coursework, fulfillment of competency requirements, and management of the resident's time? Many universities have software provided by the Graduate Medical Office intended for use by physician residency programs. However, these tools are often unavailable or unsuitable for non‐physician programs. For example, the reporting requirements of physician residency programs often add substantial complexity and costly administrative training requirements to utilize such software tools. In addition, they may not be accessible from remote locations. What is required is a Web‐based Application Service Provider (ASP) software package that provides a suitable database and interface, with regular backup to remote secure servers.

The solution proposed is to manage the residency program using a commercial ASP software solution. Four potential application solutions were evaluated: MyResidency 2.0 from eResidency (Challenger Corporation, Memphis, TN); E*Value Allied Health Solution from Advanced Informatics LLC (Minneapolis, MN); Residency Management Suite from New Innovations, Inc. (Uniontown, OH), and Allied Health Student Tracking System from Typhon Group, LLC (Metairie, Louisiana). This article describes the criteria, summarizes the features of four software tools, and provides a detailed analysis of the selected tool.

## II. MATERIALS AND METHODS

Some desirable components of an accredited residency software package include tracking modules for:

- Administration – Tracking of annual TB test records and immunizations, CPR training, and other employment requirements is an essential component of program management. The tool should be capable of local and remote site administration with assignment of remotely assigned residents, mentors and site administrators.
- Clinical Competency Records – Procedure logs are defined as procedures performed on specific types of equipment and may include commissioning, continuing quality assurance, and other testing procedures. Case Logs are linked to specific patients and presentations.
- Conferences – Routinely scheduled clinical conferences are usually not graded but attendance, lecturer and content should be recorded.
- Courses – Courses may be short (immersion, 3–5 days) or long (full semester). The course may include tests and grades and, if this is the case, grades should be recorded in a permanent record module. Courses may be offered by professional societies, vendors or other institutions. Courses may be offered onsite, remotely through on‐line training, or at remote sites. All information regarding courses should be accommodated within the application.
- Evaluations – The application should be able to email surveys automatically, and follow up if the surveys are not returned. Evaluation types should include resident by mentors, mentor by resident, course by resident, rotation by resident, and other custom forms as required.
- Student Performance Reports – The application should be able to report an accounting of time spent on the various residency tasks by each resident. In addition, there should be a complete reporting of competency completions, attendance at conferences, participation in each course and lecture, and examination performance.
- Calendar & Scheduling – The application should allow for daily, monthly or rotation resident and mentor assignments, as well as the assignment of special responsibilities or events.
- Submitted Projects and Reports – The application should allow for uploading of required projects by the resident, such as shielding evaluations or equipment performance reports. In addition, the tool should allow mentors or program directors to permanently upload narrative evaluations or disciplinary reports, with access limited to those with rights to see these documents.
- Web Features – Residents may wish to build a portfolio of experience accessible to potential future employers. Having the capability to prepare this portfolio as part of the residency management system is a desirable feature.
- Ease of Use – The tool should be intuitive and relatively simple to use as there are always new residents and mentors that will need to use the software package. Video training support for the tool is a desirable feature.
- Cost – These applications are priced by the cost of setup, annual support fees, and the number of active students. It is particularly important that the cost is affordable for small programs (2–4 residents).

Ideally, a management tool would be able to record and document each of these components and component types within the residency program. A search was therefore conducted to evaluate potential physics residency management tools. The selection criteria for evaluation were:

- The software package should be offered through an ASP with the application, user interface, and database maintained by the provider.
- Backup support from the vendor should be daily, with backup to secure servers in a remote location.
- It should have secure log‐in access for several categories of users: residents, mentors, site managers, and program managers.
- The software package must be customized, fielded, maintained, updated and archived by the user.
- It should be highly customizable respecting the components of the program and those components should reflect requirements of AAPM Report 90.^(^ ^4^ ^)^
- It should provide a complete record of the resident's experience within the program as a benefit to program reviewers.

Additionally, there are some complex relationships within the structure of a residency program. A competency may be associated with a course, rotation or conference. A course may be associated with a conference. Any of these may be associated with specific mentors, patients or machines. For each event type, the associations, the mentors, the patients and the equipment must be captured on an ongoing basis to provide proper documentation for the residency program. These can be onerous tasks.

All of the packages offered relative ease of setup and customization, and all allowed updating of data, tracking resident progress, and the capability to provide custom time, course, competency and conference reports. After comparing the features offered, we determined that cost was our most important criteria, with a simple and intuitive user interface an additional criteria. We wished to avoid unnecessary complications and unneeded features. Our program is small with only two residents in concurrent training. We experimented with demonstration versions of the software packages, and explored what customization features were available. We interacted with individuals who provide front‐line support for these packages.

Each of the packages reviewed allowed for full customization of an individual residency program; we customized the program for six‐month semesters. Ours is a four‐semester program with duration of two years. During each semester, each resident is required to complete one full academic course, with tests and a final examination. The four courses are Dosimetry, Radiobiology, Physics and Advanced Physics. Course records including attendance, grades and performance on oral examinations are accommodated within each of these packages. Other courses, such as orientation, short courses, laboratory courses, vendor courses, board review courses, self‐study, on‐line, and other course information and records are also provided as a part of our residency program. Conferences, such as chart rounds, morbidity and mortality, journal club, tumor, multi‐modality and core competency conferences are also included as part of our program.

During each semester, the resident is expected to complete the individual competencies within three of a total twelve competency sections. Each competency section contains a number of individual competencies that must be completed. The competency sections are to be completed in order. Competency sections include external beam treatment planning and verification, brachytherapy treatment planning and verification, room shielding design, quality assurance (daily, monthly), annual calibrations of clinical equipment, TBI photons and TSE electrons, intraoperative electrons, stereotactic cranial and body, IMRT / IGRT, Respiratory gating, HDR / LDR Brachytherapy, and administrative and professional duties. There are a total of approximately 150 individual competencies in our program. The software packages typically allowed for 1000 or more competencies to be entered, many more than required by our program. All packages allowed for individual competencies to be grouped together into sections.

The applications listed typically serve dozens or hundreds of residency programs and serve many thousands of students or residents. They provide a full assortment of features, functions and flexibility beyond the needs of most physics residency programs. Any of the applications would likely provide all the functionality required to manage data for a CAMPEP residency program. In the final analysis, one application provided a superior price to performance value for our small program, and consequently was selected for implementation.

## III. RESULTS & DISCUSSION

Each of the four applications selected for evaluation meet the general criteria listed above. The applications were evaluated and the results are reported in Table 1. The application selected is provided by Typhon Group, LLC, of Metairie, Louisiana. The application most appropriate for a therapy physics residency program is the Allied Health Student Tracking module.

**Table 1:** Residency management application features.

	*MyResidency 2.0*	E*Value Allied Health	*Residency Management Suite*	*Allied Health Student Tracking*
**Administration**				
TB test records	Y	Y	Y	Y
CPR training	Y	Y	Y	Y
Immunizations	Y	Y	Y	Y
Remote site administration	Y	Y	Y	Y
Assignment of remote mentors	Y	Y	Y	Y
Assignment of remote site administrators	Y	Y	Y	Y
**Clinical Competency Records**				
Competency Procedure Logs	Y	Y	Y	Y
Competency Case Logs	Y	Y	Y	Y
**Conferences**				
Event Attendance	Y	Y	Y	Y
Lecturer and Content	Y	Y	Y	Y
**Courses**				
Course Grade Module	Y	Y^a^	Y^a^	Y^a^
Attendance Module	Y	Y	Y	Y
Online Training Module	Y	Y	Y	Y
Vendor Training Module	Y	Y	Y	Y
**Evaluations**				
Customizable Forms	Y	Y	Y	Y
Automatic Distribution	Y	Y	Y	Y
**Student Performance Reports**				
Competency Completion	Y	Y	Y	Y
Conference Attendance	Y	Y	Y	Y
Coursework Records	Y	Y	Y	Y
Time Records	Y	Y	Y	Y
**Calendar & Scheduling**				
Daily assignments	Y	Y	Y	Y
Rotation & Mentor Assignments	Y	Y	Y	Y
Rotation & Clinic Assignments	Y	Y	Y	Y
Special Events	Y	Y	Y	Y
**Submitted Projects and Reports**				
Submissions from Students	Y	Y	Y	Y
Submissions from Mentors	Y	Y	Y	Y
**Web Features**				
Program Description	Y	N^b^	N^b^	N^b^
Online Student Portfolio	Y	Y	Y	Y
**Cost**				
Initial set‐up		$1000	$250 ‐ $500	$250
Annual system support after year 1	$1000 ‐ $2000	$1000	$750^c^	$250
Each student annually		$95^d^	$0	$75
Annual cost: 2 / 4 students	$1000 ‐ $2000	$1000 / $1000	$750 / $750	$400 / $550

a Course grades may be recorded in an evaluation module that queries test scores and final grades for each course.

b May be set up by registering a sample resident and using the website assigned for the resident portfolio.

c Includes fee for up to 15 residents annually.

d The $1000 annual fee includes registration for 9 residents ($950).

**Figure 1:**
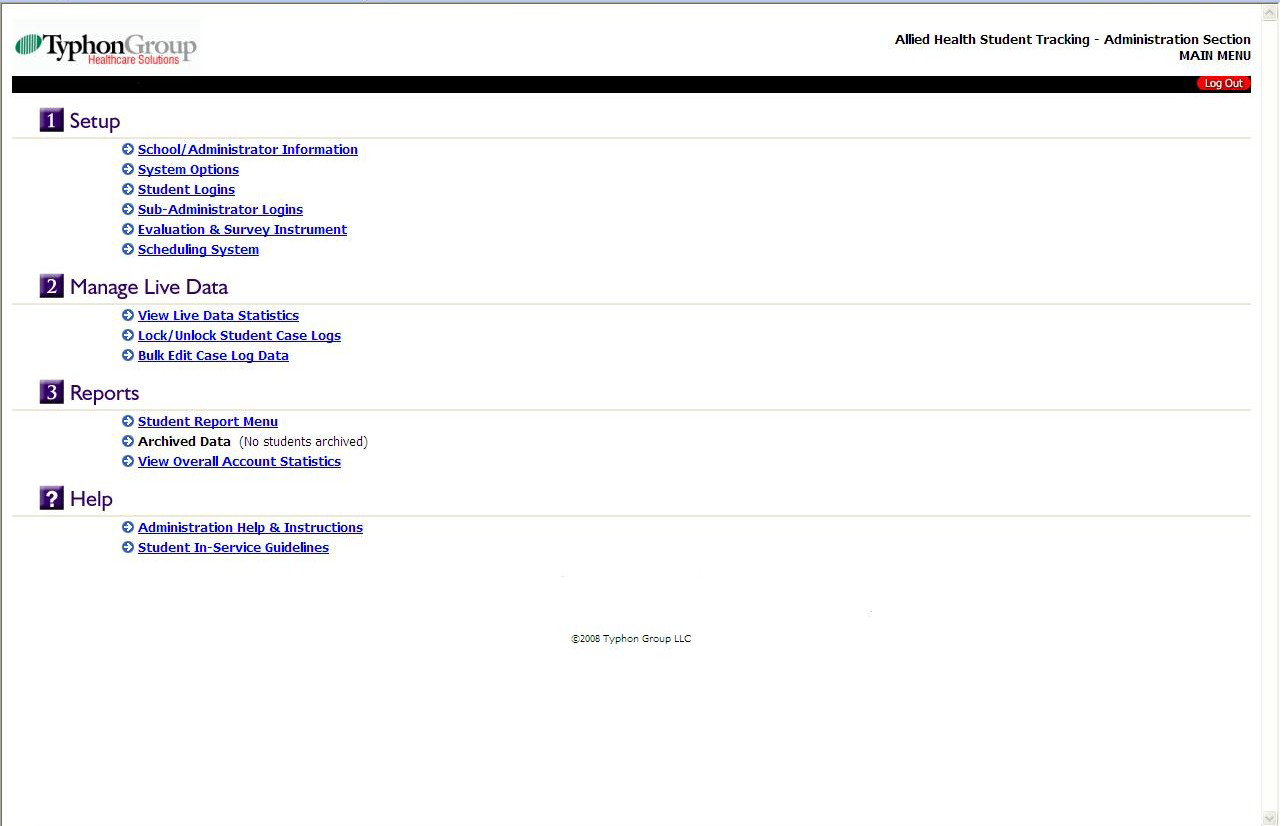
This is the main operational screen of the Typhon Group Allied Health Student Tracking module for the program administrator. Only the program administrator has access to the setup features in section 1. (All personal or institutional information was eliminated from every figure for review purposes.)

The login screen leads the program manager to the main operational screen of the Typhon Group ‐ Allied Health Student Tracking module, reproduced in Fig. 1. This page contains four sections: a) Setup Page, b) Manage Live Data, c) Reports, and d) Help. Each of these will be discussed in the following sections.

### Setting up the software tool for initial use

**Figure 2:**
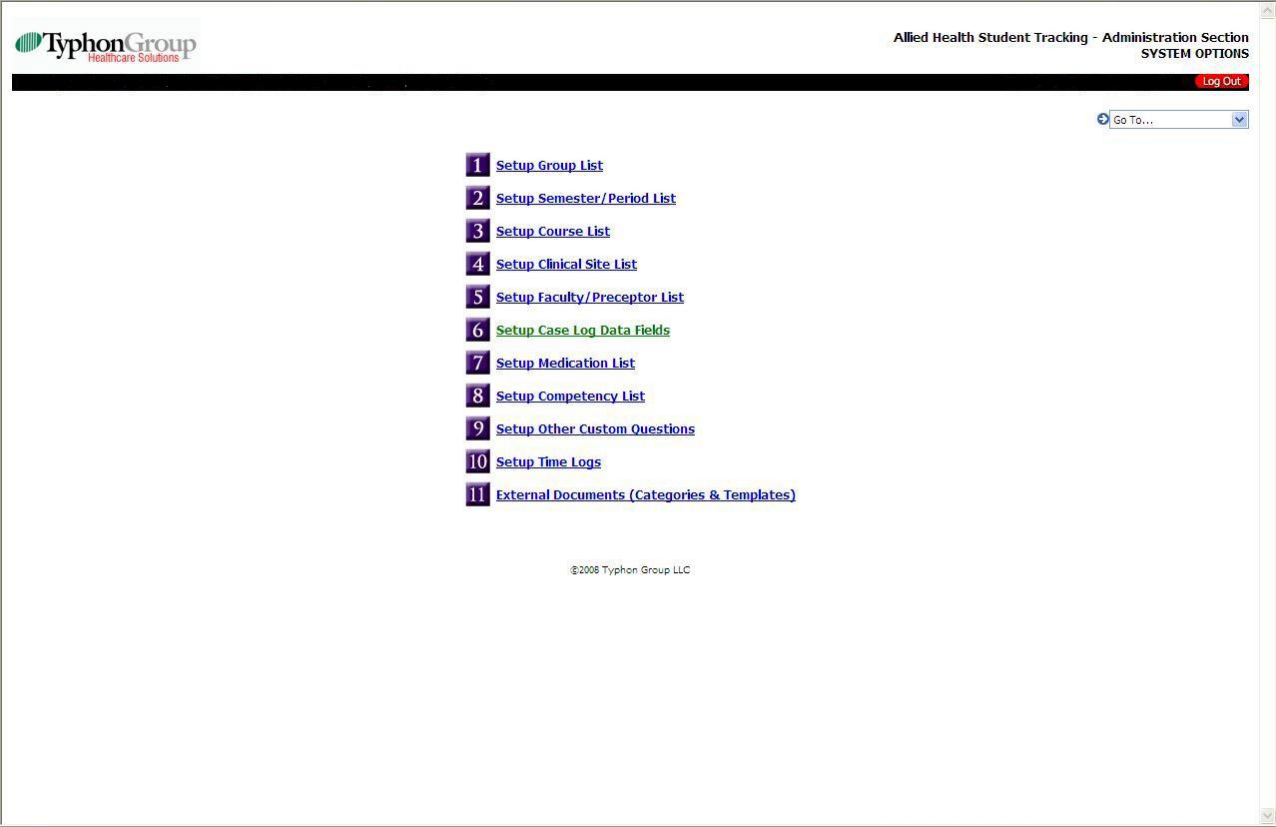
This is the main program configuration screen for the Typhon Group software, corresponding to selecting System Options under Setup from Fig. 1. The medication list is usually not configured for therapy residency programs.

The “System Options” section allows for detailed customization of the residency program (Fig. 2). The following comments discuss some of the section's important features. The “Setup Group List” allows students from different categories to be specified: residents, MS students, PhD students, DMP students, etc. The “Semester/Period List” allows for selection of rotation length: quarter, trimester, semester, etc., and for periods of 3, 4 or 6 months, respectively. The “Setup Course List” provides for the listing of type of course within the program. Orientation, traditional, laboratory, on‐line, vendor training, short, conference‐based, structured self‐study, and other courses may be listed. The “Clinical Site List” allows for specification of remote clinical sites, site coordinator contact and demographic information for affiliated program sites. The “Faculty/Preceptor List” provides demographic information for mentors including the associated clinical site, and includes credentials, specialty, board certification and contact information. The “Case Log Data Screen – Field Settings” section specifies the range of information recorded for a student activity encounter. These data include Date Control (how far in the past a student is allowed to record an activity) and Competency Categories (twenty are allowed). Competency Categories may be generally associated with a specific quarter or semester within the program. The “Setup Competency List” section lists each Competency Category, which may be associated with a specific quarter, trimester or semester. Within each Competency Category individual competencies are listed; up to 50 competencies per Competency Category may be specified. Table 2 contains a sample list of competencies under the Competency Category: Annual Calibration of Clinical Equipment. Competencies may be associated with a specific patient or equipment encounter, course, semester or conference. Various categories of time such as conference time, encounter time, course time, sick hours/days, vacation days, chart review time, and comp time may be recorded. Up to twenty categories of time log items may be listed. External Document categories are listed for documents up to 500 kb provided by residents to the program director as part of the program. Examples include shielding calculations, special medical physics reports, and PowerPoint presentations for conferences or physician resident teaching encounters.

- Student Logins: This category lists login and other demographic information for each resident.
- Sub‐Administrator Logins: The demographic data for the site coordinator of an affiliated program may be listed here along with data permissions and any administrative responsibilities permitted.
- Evaluation and Survey Instrument: Resident evaluation, course evaluation, program evaluation and mentor evaluations surveys may be created, managed and conducted using this tool.
- Scheduling System: This tool allows the program manager to add, edit and delete events from the schedule of a resident or several residents. Residents may enter or request for approval their own events with permission.

**Table 2:** Sample list of competencies.

Annual Calibration of Clinical Equipment
Annual Simulator Calibration
Annual CT‐Simulator Calibration
Annual Linear Accelerator Calibration
Annual Intraoperative Linear Accelerator Calibration
Annual Instrument Inter‐comparison
TG‐51 Photon Calibration
TG‐51 Electron Calibration
Annual TomoTherapy Hi Art Unit Calibration
Operation of Linear Accelerators
Operation of TomoTherapy Unit
Operation of Farmer type Chamber/Electrometer
Operation of Well type Chamber/Electrometer
Operation of 3‐D Scanner
Operation of Unfors Radiographic Meter
Operation of Unfors CT Meter
Operation of Intraoperative Unit

### Managing live data to view resident activities

This section records and displays statistics based on live records such as patient encounters, course and conference attendance, and completed competencies. A security feature allows the program manager the ability to prevent data in the system from being altered. Data may be locked or unlocked by selecting a specific date and range.

### Generating resident reports to view resident progress

**Figure 3:**
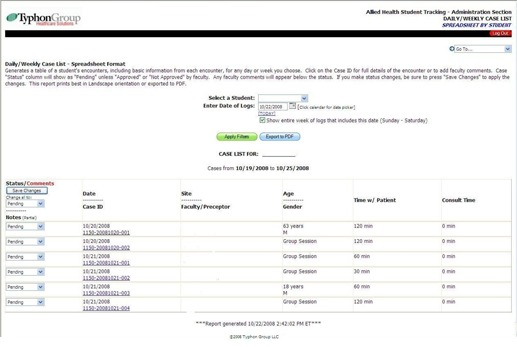
The Daily/Weekly Case List Spreadsheet Format displays a table of the resident's encounters for any day or week selected. Clicking on the case number shows the full details of the encounter and allows for faculty or mentor comments to be recorded. Competencies are initially “Pending”, and may be selected “Pending”, “Approved”, or “Not Approved” within the software. (Faculty/Preceptor information was initially present, but removed for privacy and for the purposes of peer review.)

The application provides a number of alternatives for viewing the progress of the resident through case log reports. One very useful display is the Daily/Weekly Case List (Spreadsheet by Student) (Fig. 3). This option generates a table of a student's encounters, including basic information from each encounter, for any day or week chosen. Patient encounters, competencies, attendance or lectures may be evaluated. The status is initially shown as “Pending” until “Approved” or “Not Approved” by faculty. The completion of a competency may be listed by the resident; completed competencies are displayed in blue, those not completed are displayed in red. Competencies are listed as Observed, Assisted or Done. The program manager may require a minimum number of cases to be “Done” for each competency.

Attendance at a course lecture or conference is recorded by the resident. Information includes the lecturer, topic, date and time of lecture, duration, and any associated course. Resident submissions such as shielding reports, special medical physics reports or PowerPoint presentations may be reviewed with faculty/preceptor comments. Students may create an on‐line portfolio of resident accomplishments and experiences along with a detailed Curriculum Vitae and a photograph. This portfolio is assigned a Web address for use by the resident in a job search.

An active file within the system is maintained for all current residents and purchased one year at a time. All records associated with a specific resident may be locked and archived upon graduation or a short time thereafter, with no additional cost to the program. However, the records are still accessible and may be viewed at any time by the program manager for up to three years after graduation. Program managers should print out and/or electronically record in a separate permanent local database all resident information needed for future uses, such as program reviews. Otherwise, the ASP database would become larger than needed and much more expensive to maintain. In our view, it is reasonable for program reviewers to view paper or Adobe .pdf electronic archives for previous residents and electronic records for current residents.

### Support and help functions provided with the software tool

Complete instructions for managing the application are provided. Several hours of video instruction are included in this section, broken up into segments related to specific topics for specific persons (program administrators, mentors and residents). Common topical questions are addressed in the Administrative Frequently Asked Questions (FAQ) section. A simple one‐page instruction sheet to guide residents in this use of the application is offered.

### Experience with the system

Although the application seems complex at first, the menus are well laid‐out and intuitive to follow. Our program of semesters, courses, conferences, competencies, evaluations and examinations fit readily into the overall structure of the software package. We viewed the tutorials and defined the scope of information required by the system. We listed the courses, named the conferences, defined the competency categories and listed the individual competencies within these categories. Also we listed the demographic information for mentors in the program: roles, contact information and credentials. With telephone help and support from the vendor, the system was ready for initial testing after approximately four hours of setup. The system was completed with approximately another four hours of program entry. This included entry of all demographic information, faculty, residents, conferences, courses, competency categories, individual competencies, course descriptions, and evaluation and survey instruments. We experimented with the system for about a week before releasing it to the residents and mentors. We have made only a very few changes since its release.

Residents began using the system with a lot of enthusiasm. It was easy for them to see the benefit of having all their effort in the residency program captured and recorded for archive. We released the software package the end of the first week in July of 2008, coinciding with the midpoint of the program of our senior resident and the beginning of the program respecting our junior resident. The software allows data to be entered for up to a maximum of one year in the past. This allowed our senior resident to record the entire residency experience within the software package. By December 2008, the senior resident, 1.5 years into the program, had accumulated 625 conference and course hours, 177 specific case encounters, and completed 145 competencies. In addition, 33 separate evaluation records (mentors, courses, program and resident evaluations) are included in this resident's record. The residents continue to use and value the system; the information contained helps them to build a website and portfolio of personal accomplishments, useful in their search for employment after completion of the residency.

There are some distinguishing features of a therapy physics residency program that are unique, making the use of a generic software package somewhat awkward. For example, other medical and allied health residency programs are very much oriented toward patient interaction, patient education, drug administration and patient demographics. Examples of the latter are rural population, indigent populations, and Medicare or Medicaid coverage. There is some ability within the software package to limit or eliminate the queries for this type of information for a patient encounter.

There is no category for an “equipment encounter” in the software model. As therapy physics programs need to record encounters such as commissioning, annual calibrations, and chamber and electrometer monthly quality assurance, for example, there is a need to record an encounter that is not specific to an individual patient. Within this system, “equipment encounters” are listed as “group encounters”; this method has proven workable to record equipment specific tasks and competencies.

Another unique feature of medical physics residency programs is that not all of them begin on the same day, such as July 1. In addition, it is sometimes desirable to admit a resident into a program earlier or later, by up to several months. A variable start and/or finish date should not be a problem for this software system. Since courses, competencies and conferences are handled both independently and yet with optional association with one another, the system is able to handle any form of data entry at whatever point within the program. A resident may graduate at any point, assuming program requirements for coursework, conferences and competencies are completed and evaluations are satisfactory.

While this residency program does not yet have an active affiliation, an affiliation was simulated within the system using the sample student. Recording of competencies, courses, conferences and case logs for the sample resident was seamless and straightforward within the system. For this test, the approval of competencies, review of performances, and grades is handled as for any resident in the program. Completion of competencies may be approved by a local administrator within the system, if these rights are allocated. Recording of course examination results, grades and oral exam evaluations is accomplished using the Evaluation and Management feature. It is straightforward to build a questionnaire for the resident that queries the grade for each test, course and oral examination. This has proven to be a workable method for faculty to record grades of students for archiving and for subsequent program reviews. Also, it is possible to upload documents associated with a specific student but not visible to that student. This is useful if a mentor or program director wishes to deposit a narrative report on resident performance into the permanent record of the resident. This feature may serve to document disciplinary actions or probations respecting the behavior or performance of the resident. Overall, the Typhon system was deemed an appropriate tool for the management of remote or affiliated residency programs.

## IV. CONCLUSIONS

The ABR year 2014 initiative will require affiliated training for medical physics residency programs; at present there is little alternative to this eventuality. It is expected that these programs will need to train a minimum of 125 and ideally 200 radiation oncology physicists in CAMPEP‐accredited residency programs each year after 2014.

The Typhon Group software tools and database will allow tracking of resident case reports, coursework, competencies and time within a residency program. As an application service provider tool, the Typhon Group software may be used simultaneously by any number of clinical sites for a given program. Residency software and management tools are an important component if the medical physics profession is to meet the projected needs for qualified medical physicists in future years.
